# Precise surface engineering: Leveraging chemical vapor deposition for enhanced biocompatibility and durability in biomedical implants

**DOI:** 10.1016/j.heliyon.2024.e37976

**Published:** 2024-09-16

**Authors:** Tasfia Saba, Khondoker Safin Kaosar Saad, Adib Bin Rashid

**Affiliations:** a Department of Industrial and Production Engineering, Military Institute of Science and Technology (MIST), Dhaka, 1216, Bangladesh; b Department of Mechanical Engineering, Military Institute of Science and Technology (MIST), Dhaka, 1216, Bangladesh

**Keywords:** Chemical vapor deposition, Biocompatibility, Biomedical implant, Surface modification

## Abstract

Biomedical implants have revolutionized modern medicine, providing diverse treatment options for various medical conditions. Ensuring the long-term success of certain materials used in various applications requires careful consideration of their ability to interact with biological systems and withstand harsh biological conditions. Optimizing surface properties is crucial for successfully integrating biomedical implants into the human body, ensuring biocompatibility, durability, and functionality. Chemical Vapor Deposition (CVD) has become a crucial technology in surface engineering, offering a precise technique for applying thin films with customized properties. This article provides a comprehensive study of surface engineering for biomedical implants, specifically emphasizing the CVD coating technique. By carefully manipulating chemical reactions in the vapor phase, CVD allows for the creation of coatings that enhance wear resistance, minimize friction, and improve biocompatibility. This review also explores the underlying principles of CVD, the various process parameters involved, and the subsequent enhancements in implant performance. Using case studies and experimental findings, it showcases the ability of CVD to greatly enhance the durability and effectiveness of biomedical implants.

## 1 Introduction

The subject of biomaterial science presents a unique and unprecedented chance to enhance and preserve the lives of millions of people globally. Combining chemistry, biology, engineering, medicine, and biomaterial science results in innovative materials to address medical issues. Many medical implant materials have been developed and used more often in recent years [1]. Due to growing public concern for health and an aging population, there is a growing need for affordable, reliable, and secure biomedical equipment worldwide. Biomedical devices are essential in the healthcare business for diagnosing, evaluating, and treating a broad range of illnesses and injuries affecting different areas of the body [2].

The field of biomedical science has historically depended on applying natural or synthetic materials to fabricate biological structures for implantation. This practice has profoundly impacted the quality of life for individuals who have experienced the loss or dysfunction of biological components [3]. The increasing existence of age-related conditions, namely arthritis and joint pain, has resulted in a heightened need for biomedical devices incorporating artificial components. These devices aim to provide remedies by replacing impaired or diseased tissues. The prevalence of chronic diseases in industrialized countries is significantly higher among those aged 50 and above, constituting approximately 50 % of all cases [4].

Consequently, there has been a prominent increase in research efforts converging on biologically functional materials. Degenerative disorders, such as arthritis, can induce joint discomfort and dysfunction by modifying the mechanical characteristics of bones due to excessive stress or the body's constrained capacity for self-repair. With the ongoing growth of the elderly population, it is projected that almost 90 % of adults aged 40 and beyond will experience the presence of one or several degenerative diseases. Thin-film deposition is a crucial process in the landscape of modern technological breakthroughs, especially in the electronic and medicinal sectors. Chemical Vapor Deposition (CVD) has quickly become a go-to process among the several available for fabricating the devices and structures that make up the microelectronics and biomedical implants industries. To ensure optimal performance and biocompatibility, medical equipment frequently demands the application of specialized coatings. These devices' complex shapes and tight tolerances need coating methods that provide uniform surface coverage. Chemical Vapor Deposition (CVD) has become a popular option because of its capacity to produce thin films that are both thick and highly pure, two characteristics that are essential for the aforementioned uses [5].

In 1969, Krupp, a company based in West Germany, used thermal Chemical vapor deposition (CVD) techniques to deposit a titanium carbide (TiC) film onto carbide cutting inserts, marking the first application of chemical vapor deposition (CVD) for coating cutting inserts. Japan and other regions saw increased competition due to this technological development. Sumitomo Electric improved traditional production methods in 1976 by adding a special resistant surface layer to a cemented carbide substrate; this led to a theatrical increase in CVD-coated inserts. CVD is a sophisticated process in which gaseous reactants dissociate and interact chemically in a high-energy (heat, light, or plasma) environment. The result of this laborious procedure generates stable and solid items. Deposition of powders or films relies heavily on both gas-phase homogeneous chemical reactions and surface-based heterogeneous chemical reactions [6].

The demand for biomedical devices made of artificial components to replace damaged tissues has increased due to the growing incidence of circumstances like arthritis and joint discomfort among older individuals, as well as the prevalence of loss or impairment of natural or synthetic biomedical components. In wealthy countries, they make up half of all chronic disorders affecting adults over 50 [4]. Therefore, there has been a lot of recent interest in biologically functioning materials. Joint pain and lack of mobility are common complaints among those with arthritis and other degenerative diseases. Degenerative diseases alter bone mechanical properties due to abnormal loading or a failure of the body's biotic self-healing system. The rate of occurrence of these degenerative illnesses has been steadily increasing over the last several decades, and it is believed that 90 % of adults over the age of 40 are affected by these.

According to A Tempieri et al. [7], The body's natural capacity to heal declines with age, so it's important to find ways to enhance and sustain tissue regeneration after damage or sickness. One promising approach is to use innovative biomaterials and devices that regulate cell fate. The new solution must induce biomimetic stimuli on stem cells by mimicking the physiological extracellular environment. This is necessary to get satisfying outcomes even when the regeneration process is tougher and longer due to cell senescence, which is common in the elderly [7].

The two most common reasons for implant failure are mechanical and biological factors. The magnitude of the load applied to the implant depends on the body's positioning during walking. The hip joint experiences a load almost four times the weight of a human, whereas the knee joint bears a load nearly three times the individual's body weight [8]. Strenuous physical activities, such as cycling, leaping, or running, exert additional stress on the implant, potentially resulting in technical malfunction [9].

When used in a clinical setting, inflammation responses caused by an improper implant surface might amplify the inflammatory response the surgery has set in motion. Then, for instance, blood clots may develop around a stent, or non-mechanically stable connective tissue may grow around a bone implant (such as a dental implant, an artificial hip joint, etc.). A process of bone regeneration and blood flow comparable to that without an implant may result from less physiologically disruptive surfaces. One of the primary aims of biomaterials research is the design of such surfaces using diverse methodologies [10].

When the human body reacts to external substances, the implant loses its biological function because the body's defenses are disrupted. In the long run, this causes significant hardship for the person concerned. Artificial stress and a dynamic environment are used to test implant compatibility. Patients who undergo numerous medical procedures only to have their bodies reject them may experience severe psychological suffering if their attempts at organ transplantation fail [11].

Various materials, such as metals, polymers, ceramics, hydroxyapatite (ceramic), natural materials, polyurethane, polyethylene, and polyimide [12], are used to construct medical devices that come into contact with biological media. New metallic alloys are being developed for use in biomedical applications to create structural materials that are highly biocompatible in terms of biological, chemical, and mechanical factors. This is in response to the growing demand for implants in younger patients and the need to extend the average human life expectancy. Because of their great mechanical characteristics, such as a low Young's modulus, several Ti-alloys were created using biocompatible elements, including Zr, Ti, Nb, Ta, and Mo [13]. Materials used in orthopedics, cardiovascular stents, surfaces that prevent bacterial growth, tissue engineering, drug delivery systems, and biosensors. Enhanced biocompatibility, antibacterial, antiwear, and properties corrosion protection can be added to various substrates for biomedical applications using CVD coatings [14].

Osseointegration of native tissue with implant materials is a challenging phenomenon that is difficult to achieve. As a promising method for achieving the ideal surface properties in implant materials concerning biological, mechanical, and other multifunctional properties, suitable bioactive coatings, such as biocompatible coatings, antimicrobial coatings, osteoconductive coatings, hard coatings, coatings for sustainable antimicrobial paints in clinical environments, antibiotic release, and corrosion resistant coatings, are examples of the types of coatings that can be used [15].

This review proposes a thorough examination of different CVD coatings, including the materials employed, the qualities of the implants, their applications, limitations, and potential future advancements. This paper offers a summary of different Chemical Vapor Deposition processes where different coating materials and their distinct mechanical and physical properties were also studied. The widely used CVD method makes it easy to coat a variety of substrates, including biomedical implants. Coating substrates with CVD significantly improves the performance and properties of biomedical implants, including enhanced biocompatibility, wear resistance, hardness, durability, drug delivery capabilities, and resistance to corrosion and oxidation. Medical implants, including orthopedic, dental, cardiovascular, neurological, and drug-eluting implants, use CVD coating for various applications.

## 2 Principles of chemical vapor deposition (CVD)

Numerous industries use CVD coatings on various items for the general public. The durable and environmentally friendly thin films used to make these surfaces are highly sought after. The high-performance thin film chemical vapor deposition (CVD) technique is widely utilized in diverse applications, including instruments, wear components, analytical flow path components, and machine tools. CVD is superior to other methods regarding conformality or a film's ability to coat a substrate with a uniform texture. Thin-film coating is the most significant process for producing many biomedical gadgets. Owing to the deposition process, coatings can improve the performance of materials. It is very useful for manufacturers and engineers to select appropriate parameters to acquire the best hardness and reduce time and cost when they have a reliable and accurate model that can predict the hardness of CVD processes [16].

CVD can achieve a uniform material layer deposition on all implant surfaces, encompassing inner and outer surfaces. Coatings with superior purity can be made by employing gaseous raw components of exceptionally high quality. The circumstances of the deposition can be adjusted to alter the coating's shape and surface area. CVD is a tried-and-true process in which a volatile precursor reacts inside a chamber normally held under vacuum conditions. The temperature within the chamber is raised deliberately to kick off a chemical reaction. This leads to the precursor gas's breakdown and the required coating's formation. This coating will cling to whatever it comes in contact with. Over time, coating material builds up on the surface, generating a coating that eventually completely covers the open area. The hardness of the coating varies with its texture; films that are substantially orientated have a lower hardness than coatings that are not structured. The textured films' reduced grain size contributes to their increased toughness and improved wear resistance, regardless of surface roughness [17].

**Fig. 1:**
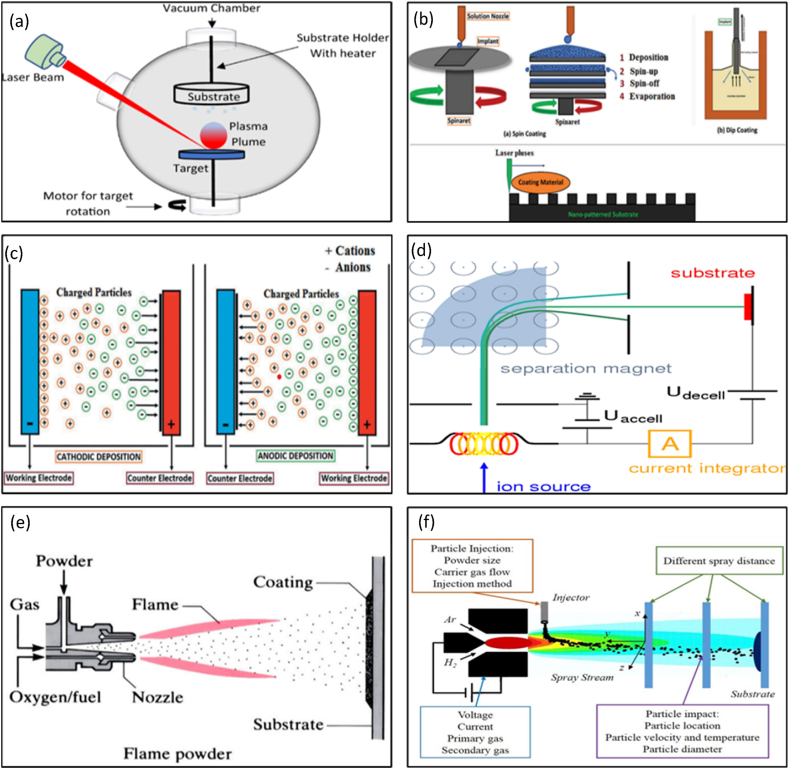
Different coatings: a) Pulsed laser deposition; b) So-gel deposition; c) Electrophoretic Deposition (EPD); d) Ion Beam Deposition (IBD); e) Magnetic Sputtering; f) Thermal Spraying Process [19,20].

The principles of numerous CVD coating techniques are dissipated in Fig. 1. Pulsed Laser Deposition (PLD) (Fig. 1(a)) occurs when a laser vaporizes a target material, which is then deposited onto a surface or substrate. Ion-beam deposition allows for forming ultra-pure epitaxial thin films at low temperatures, yielding film properties that cannot be achieved using more conventional deposition methods [18]. To create a sol-gel coating, solid particles (1–500 nm) are suspended in a liquid solution and sprayed, dipped, spun, or doctor-bladed onto a substrate (Fig. 1(b)). The gel is subjected to incineration or drying processes until it transforms into a thin layer covering the substrate's surface. Layers of calcium phosphate are formed by immersing a metal specimen in a low-temperature calcium and phosphorus gel. Since the resulting coating is porous and less thick, it must be calcined at temperatures between 400 and 600 °C before it can be used. The bonding strength may be increased by depositing several layers of the covering material over the implant.

Thin films, coatings, and composite materials are often produced using the wet deposition technique of electrophoretic deposition (EPD) (Fig. 1(c)), also known as electrophoretic coating or electrocoating. Electrophoretic deposition is a technique that capitalizes on the phenomenon of charged particles in suspension undergoing movement in response to an externally supplied electric field. Consolidating these particles into films, which may be cast onto any substrate or into thick, bulk components, is made possible by the electric field [18].

Compared to conventional coating techniques, Ion Beam Deposition (IBD) (Fig. 1(d)) can produce bio-coatings with significantly stronger adhesives. Ion bombardment helps the substrate and coating atoms interact, producing high adhesive strength. This causes the substrate-coating contact to have an atomically mixed zone. The IBD process is very reliable and can be performed at a low substrate temperature without causing any negative changes to the characteristics of the bulk substrate. Moreover, the method offers better control over the chemical composition and coating microstructure [21].

Magnetic sputtering (Fig. 1(e)) is a technique where the material is ejected onto a "substrate," such as a silicon wafer, from a source called a "target." Using a gas plasma (argon, neon, krypton, or xenon), material from a negatively charged target is removed during testing, and Research has demonstrated that Radiofrequency magneto sputtering can successfully produce small hydroxyapatite films on titanium substrates. Magnetron sputtering results in low-friction, wear-resistant, and corrosion-resistant coatings [21]. Using the heat of an ionized inert gas (plasma), ceramics or metal particles are melted during the plasma spraying (Fig. 1(f)). The surface to be coated is then sprayed with the melted powders to create a protective coating that protects against wear, corrosion, and high temperatures. The method has benefits like a quick deposition rate and cheap cost. In addition, due to the ability to keep the target at a low temperature in the plasma flame and the chemical inertness of the gas, the risk of thermal degradation of the coating and substrate is significantly reduced compared to alternative high-temperature methods. Nevertheless, poor adherence between the coatings and substrates plagues plasma-sprayed coatings, and the procedure may cause fundamental alterations in the microstructure of the coating material [21]. The implementation of different ways of CVD procedures depends on the purpose. The pros and cons of various strategies are laid forth in Table 1, making it simple to pick the best one for each given task.

**Table 1:** Pros and cons of various coatings [19].

Types of coating	Thickness	Pros	Cons
**Pulsed laser deposition**	0.05–5 μm	The coating is dense and porous. Crystalline and amorphous layers applied.	Expensive. The elevated temperature hinders the concurrent incorporation of biological agents.
**Sol-gel technique**	<1 μm	Strong adhesion to the surface	Inexpensive. Reduced thermal processing temperatures. Thin layers. A controlled environment is necessary.
**Electrophilic Deposition**	0.1–2.0 mm	Coating thickness that is uniform throughout. The deposition was made quickly.	Expensive Coatings that are devoid of cracks are hard to manufacture. High temperatures for sintering are necessary.
**Ion beam deposition**	0.05–1 μm	Powerful adhesion. Consistent layer thickness	Expensive. Make amorphous layers and coats.
**Magnetron Sputtering**	0.5–3 μm	An even coat thickness throughout. Extremely sticky. A thick, impermeable layer. Possibility of coating temperature-sensitive materials	Expensive. Amorphous coatings are deposited at low deposition rates.
**Thermal Spraying**	30–200 μm	The deposition rates are relatively high, Cost-effective	When heated to extremes, things break down. Amorphous surfaces result from fast cooling.

## 3 Coating materials

The choice of coating materials and their mechanical properties are crucial for biomedical implants. Orthopedic implants, dental implants, and vascular stents can all benefit tremendously from coatings that boost their performance and biocompatibility. The coating shouldn't trigger an immune response or cause harmful effects when in contact with living tissue. It needs to be harmless, soothing, and allergy-free. Coatings should protect the underlying implant material from deterioration when exposed to body fluids and other hostile conditions. To ensure durability over time, the coating must adhere strongly to the implant substrate and resist delamination or peeling. Coatings with antibacterial qualities can help reduce infections associated with implants. These characteristics are essential for implants that germs could contaminate. The coating material improves mechanical qualities, antibacterial activity, corrosion resistance, and biocompatibility. Chemical etching is one of the coating techniques used to enhance osseointegration and inhibit bacterial adherence [22]. Table 2 represents the coating materials, their characteristics, and their application in biomedical implants. Table 3 represents the biomaterials that are suitable for the human body.

**Table 2:** Coating materials for Biomedical implant

Coating Material	Thickness	Characteristics	Application	Reference
Titanium Nitride (TiN)	–	Low friction coefficient High chemical inertness High hardness &wear resistance Excellent corrosion resistance. Biocompatibility.	TiN coating may reduce early bacterial colonization and biofilm formation and boost fibroblast cell proliferation, attachment, and adhesion. Reduces wear and extends orthopedic and dental implants. Often used to coat joint replacement components.	[23]
Titanium dioxide (TiO_2_)_5_	500 μm	High biocompatibility and bioactivity. Promotes cell adhesion and osseointegration. Photocatalytic properties.	Antimicrobial agents that are novel and developing include TiO_2_ coating, which is used to combat human pathogenic microbes. Consider for medical implants, drug delivery and antibacterial fields. Used in orthopedic and dental implants to enhance bone integration.	[24]
Zirconium Nitride (ZrN)	800 nm	Highly crystalline Uniform microstructure Biocompatibility. Good corrosion resistance. High hardness and wear resistance	Used to strengthen dental implants and orthopedic devices. ZrN coatings on Ti preserve titanium's biocompatibility and provide antibacterial action. Biomolecular pellicles and bacteria on implant surfaces start inflammatory processes.	[25]
Diamond-like Carbon (DLC)	700 nm	Good mechanical Tribological qualities Appropriate biocompatibility Outstanding corrosion resistance High chemical inertness Less cytotoxicity and inflammation	ideal for the proliferation of cells such as osteoblasts, fibroblasts & macrophages. The hip and knee joint simulator's DLC coatings reduce wear and corrosion. Improved hemocompatibility in cardiovascular implants and biological performance, reduced platelet adhesion and activation, and limit thrombogenicity.	[26].
plasma-sprayed hydroxyapatite coatings (HACs)	200 μm	Excellent adhesion Highly biocompatible Better fixation of the implant High crystallinity Controlled porosity	Promising orthopedic implant applications and rapid bone healing. plasma-sprayed HA coatings (HACs) for alternative orthopedic implant attachment on Ti-6Al-4V showed that the new bone adhered directly to the HACs, resulting in good adherence.	[27]
Graphene	–	High thermal conductivity, Young's modulus, and electrical conductivity Anti-bacterial properties.	Investigated for use in various implants, including neural and orthopedic devices. Potential applications in biosensors and other biomedical devices.	[28]
Silicon Nitride (Si_3_N_4_)	100 nm	High fracture toughness High-strength High hardness and compressive strength Excellent wear resistance	Used in spinal implants, dental implants, and other orthopedic devices. Si_3_N_4_ may reduce aseptic loosening since it is antimicrobial and more tolerated than Ti oxide layer particles secreted in hip periprosthetic tissues.	[29]
Bioactive Glasses	100 μm	Promote bone bonding and growth. Biocompatible and bioactive. It can be doped with therapeutic ions for enhanced functionality.	Enhance host tissue integration, biological function, and bacterial prevention. Used in bone grafts and coatings for implants to enhance osseointegration.	[30,31].

**Table 3:** Application of biomaterials and their mechanical properties [19].

Biomaterial	Mechanical Properties			Application
	Tensile Strength	Hardness (Hv)	Young's Modulus (Gpa)	
**Stainless Steel**	586–1351	190	200	Knee and hip replacements; dental implants; prosthetic heart valves; artificial spinal discs; artificial hips; prosthetic shoulders.
**Titanium**	760	–	110	Hearing aids, prosthetic hips and knees, artificial heart valves and pacemakers, titanium dental implants, and orthodontic implant sutures.
**Cobalt-Chromium**	450	655–1896	220–230	Hearing aids, prosthetic hips and knees, artificial heart valves and pacemakers, titanium dental implants, orthodontic implant sutures
**Alumina**	250	2000–3000	280	Orthodontic anchors, dental implants, bridges, artificial acetabula and femoral components, spinal spacers, and extensors.
**Zirconia**	200–500	1000–3000	150–200	Hip and knee replacements; dental tendon and ligament repairs; periodontal disease restorations; bone grafts.
**Calcium Phosphate**	69–193	350	40–117	Orthopedic, face surgical, and dental implant treatments and restorations; dental fillings; jawbone rebuilding; implant coatings.

## 4 Property enhancement by CVD coating in biomedical implants

CVD is a highly adaptable method used for the deposition of thin films, and it is widely utilized in several industries, including biomedical engineering, particularly in biomedical implants. CVD is a versatile technical procedure that may modify the surface properties of biomedical implants, improving their biocompatibility, durability, and functional performance. Improving the efficacy and safety of many biomedical implants used in orthopedics, cardiology, dentistry, and other fields of medicine is a challenging task that these coatings accomplish. In addition, by enhancing implant longevity and reducing possible difficulties, the thorough study of implant performance improves patient outcomes.

**Fig. 2:**
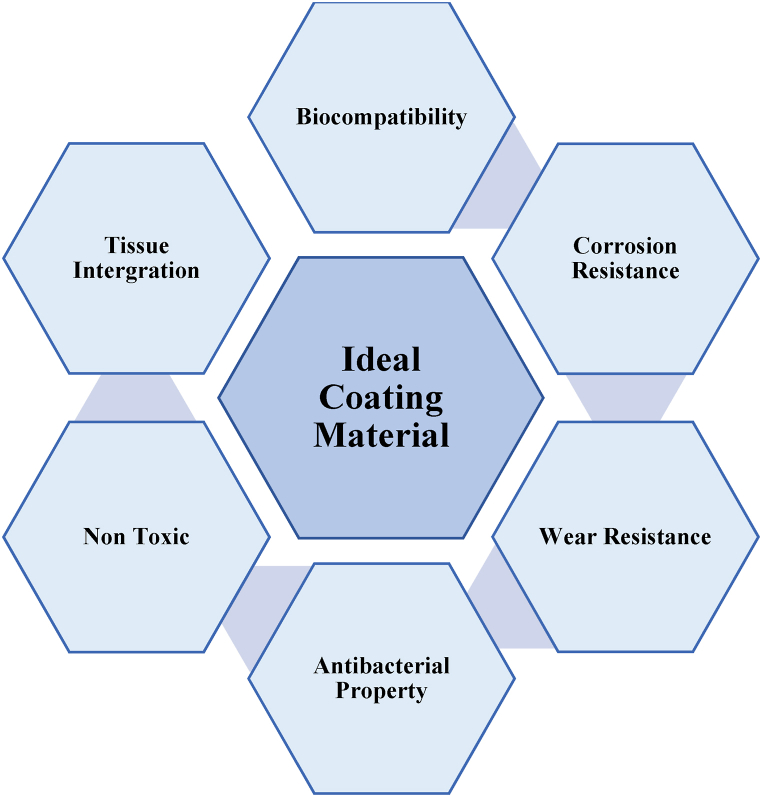
Essential Characteristics of bio implant material.

The essential characteristics of bioimplant material are displayed in Fig. 2.

### 4.1 Surface modification for enhanced biocompatibility

Surface modifications are a common way to improve the biocompatibility of materials and medical devices. Biocompatibility is the capacity of a substance to carry out its intended function inside the body without eliciting an immune response or causing damage to the surrounding tissue. A material's biocompatibility may be enhanced by altering its surface properties. Coatings are an excellent option for modifying the surface of implants because of their many benefits, such as accelerated tissue regeneration, protection against germs, and controlled waste collection. Bioactive and bioinert coatings exist, as do hydrophilic, tissue-mimicking, biodegradable, and conductive coatings [21].

#### 4.1.1 Bioactive coatings

Unfortunately, biocompatibility cannot be ensured by using surface modification approaches. Therefore, coating the bioactive surface of implants with an appropriate substance that meets the patient's demands is a viable option for modifying the bioactive surface. Over the past few decades, rapid advances in implant technology have prompted researchers to focus on creating implants for multiple purposes, each tailored to a patient's needs. Some metallic implants have severe corrosion problems, manifesting as organ and tissue irregularities in reaction to elevated ion concentrations, in contrast to their biologically similar counterparts like Tricalcium Phosphate (TCP) and Hydroxyapatite Phosphate (HAP) [21]. Titanium cobalt-chromium alloys and stainless steel-based implants are the most prevalent varieties. Although metal implants are sturdier and more stable, ceramic and polymer bio-composites perform better in bioactivity. Studies have been conducted on coating metallic implants with bio-ceramics or other apatite-based materials. Reviews of the relevant literature support the positive effects of covering metal implants with ceramic. Depending on the coating's composition and physiochemical qualities, ceramic/ceramic or ceramic/polymer composites may also be used in the coating process [21].

Implants in artificial joints eventually loosen and develop local inflammation due to metal ions (corrosion products). Corrosion, intergranular corrosion, pitting, initiation of cracks, and cracking on the surface have all been seen in patients' thighs, suggesting that the stainless-steel implants are failing [32]. According to Niinomi [33], A passivation oxide layer composed of Cr_2_O_2_ forms in the alpha phase of chromium alloys. As a result, it has remarkable corrosion resistance, especially in chloride environments. Co-Cr is a more corrosion-resistant material than stainless steel because of this passivation process. Co-Cr is utilized in whole joint replacement in the knees, hips, etc., because of its strength and fatigue/wear resistance, although the replacements are not ductile and only stretch by roughly 8 % at most. The elastic modulus of Co-Cr is 220–230 GPa, while that of bone is just 30 GPa. Cobalt-based alloys have the potential to release metal ions owing to bio-corrosion, which can have unfavorable effects on implants. Co and Ni ions, found in Co-Ni-Cr-Mo alloys, have been associated with allergic reactions [33].

#### 4.1.2 Bioinert coating

Bioinert materials are often used for tissue replacements since they are non-reactive with body fluids and tissues and have intrinsic stability within the human body. Fibrous structures frequently enclose bioinert materials after insertion into a living organism to keep them apart from the surrounding bone. Because hydroxyapatite and bioactive glasses form an apatite layer on their surfaces similar to the bone after implantation, hard tissue replacements are being utilized more frequently to enhance the connection between implants and bone tissues. This is accomplished without the production of fibrous tissues. In this context, titanium is known as bioinert in its naturally occurring surface oxide form [34].

The most popular metallic biomaterials include 316L stainless steel and cobalt-based alloys. Nickel-titanium (NiTi), Magnesium (Mg), and niobium (Nb) alloys, metallic glass, and biodegradable that may find applications in biomedical contexts [35].

Due to the high specific weight, excellent corrosion resistance, lack of allergy concerns, and highest biocompatibility among metallic biocompatible materials, some titanium alloys are receiving increased attention as biomaterials. Titanium alloys must have good corrosion resistance, high biocompatibility, and excellent mechanical properties (low Young's modulus, low density for use in orthopedics and cardiology, and cardiovascular and reconstructive purposes [36].

Titanium and titanium alloys have many uses but are also inert metals that don't promote bone cell and osteoblast proliferation. Since bacterial infections are responsible for most implant failures, several studies have focused on developing methods to improve the antibacterial power of titanium implants. The passive layer on the titanium alloy surface disappears when subjected to external force and is immersed in body fluids, making the implant more susceptible to corrosion and wear due to its relative movement with the bone [37].

Titanium and its alloys possess the capacity to mitigate the aforementioned challenges. Various surface modification techniques have enhanced implants' wear resistance and biological functions. In the past decade, coatings have been employed across several contexts to customize the properties of implant surfaces and construct entirely novel surfaces possessing distinctive attributes. With the right surface treatment methods, titanium and titanium alloys' wear resistance, corrosion resistance, and biological qualities may be selectively enhanced while maintaining the materials' favorable bulk characteristics. The biomedical sectors can employ titanium and titanium alloys more frequently if they have the right surface treatment [38]. Multiple studies have shown evidence that surface modification techniques can effectively mitigate bacterial adhesion to the substrate of an implant. Implanted biomaterials can be effectively protected against biofilm formation and efficiently rid pathogens, enhancing their overall efficacy [39].

Bioinert ceramic materials have been utilized in medical devices and implants to restore the function of damaged or degenerated organs and tissues in the human body. These materials possess exceptional chemical stability, biocompatibility, mechanical strength, and wear and corrosion resistance. Bio-ceramics are utilized as implants for reconstructing and repairing diseased or damaged parts of the musculoskeletal system [40]. Bio-ceramics are classified based on their biological response in the human body, and bioinert ceramics are one type of these bio-ceramics. Bioinert ceramics are typically described as having a biologically inert nature. These ceramics do not interact with the surrounding biological tissue or cause any noticeable reaction when implanted into a biological system [41]. Bioinert ceramics are the first generation of biomaterials commonly used for hip and knee replacements, dental implants, crowns, and more. These ceramics possess remarkable characteristics, including high mechanical properties such as tensile and compressive strength, hardness, low wear, toughness, and excellent resistance to corrosion in biological fluids [42].

Considering the mechanical characteristics of the metal is essential when developing medical implants for orthopedic and dental uses that need load bearing. Extremely high tensile strength and fatigue limit are must-haves. In terms of load-carrying capability, metallic implants outperform biomaterials made of polymeric and ceramic. Metallic implants may not be as biocompatible, long-lasting, or strong as polymeric or ceramic alternatives due to their lower elastic modulus and strength [43].

Orthopedic and dental implants made of alloys like titanium or stainless steel sometimes include hydroxyapatite as a strengthening agent. Coating the implant surface with this material makes it more biocompatible with human tissues. Conversely, the implant might sustain damage or even fail if the ceramic coating previously applied to the metal or alloy begins to delamination. Furthermore, hydroxyapatite is often used as a bone repair filler. The ceramics used in the medical industry include Titanium, Alumina (Al_2_O_3_), Zirconia (ZrO_2_), Bio-ceramic, Pyrolytic Carbon, Biodegradable Ceramics, and Calcium Phosphate ceramics [44].

**Fig. 3:**
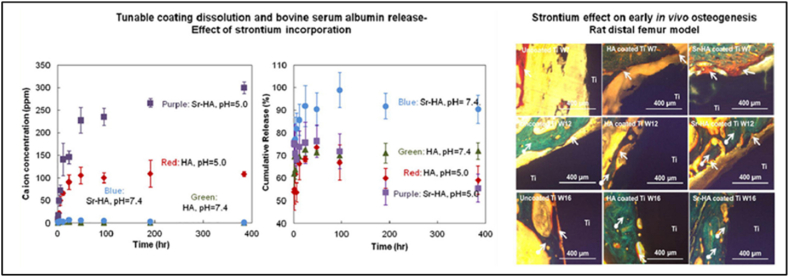
Assessment of plasma-applied HA coating for stability and bone formation [46].

**Fig. 4:**
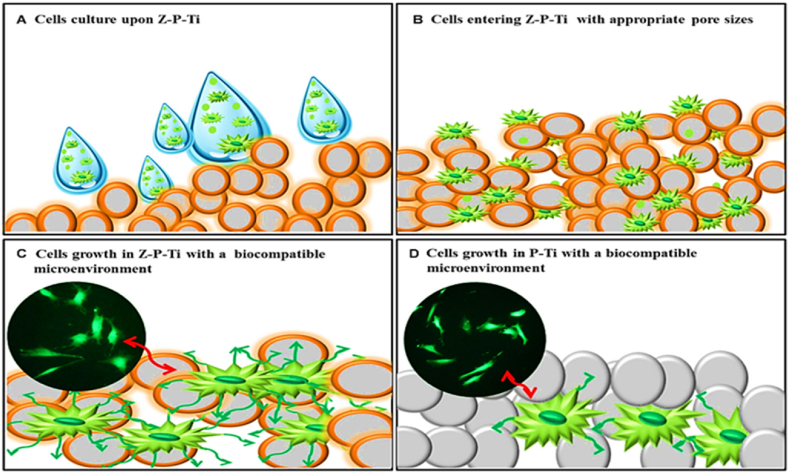
The cell surface-Z-P-Ti interaction process. Permission to reproduce Cells grow on Z-P-Ti [47].

Aluminum oxide (Al_2_O_3_) (Fig. 3) is known for its excellent biocompatibility and high resistance to wear and corrosion. On the other hand, Zirconium oxide (ZrO_2_) (Fig. 4) is commonly used in medical implants due to its exceptional durability and strong mechanical properties. The combination of mechanical properties, particularly high strength in fatigue, leads to the development of unique design and shape requirements. Alumina ceramic possesses remarkable biocompatibility, mechanical properties, and exceptional tribological behavior due to its smooth surface and surface energy [45].

Scientists can effectively address small cracks in the coating production process by carefully regulating the temperature of the plasma gas. Furthermore, the level of crystallinity reached its highest point in the environment, primarily composed of argon and nitrogen gases. Liu Y.-C. and colleagues [48] have created an innovative method called vapor-induced performing atmospheric plasma spraying (VIPF-APS) to improve the attachment and specialization of osteoblasts. A novel double-layer HA/Al2O3-SiO2 coating was suggested by Ebrahimi et al. [49], which improves monolayer HA in terms of cell behavior and biocompatibility.

Sr (Mg and Sr) doping into HA coating has been reported by Vahabzadeh et al. [46] and Cao et al. [50]. Fig. 3 represents the Assessment of plasma-applied HA coating for stability and bone formation.

Evidence of steroid synthesis in the Sr-HA coating by Ke et al. [51] indicates that this coating, compared to uncoated Ti and HA-coated implants, has a positive effect on bone regeneration pace. Assessment of Sr-HA coating-induced stability and bone growth. Visible cell adhesion demonstrated great bonding strength and good biocompatibility of (Mg, Sr)-HA on the coating's surface. Another study found that the biological and antibacterial activities may be enhanced by combining Ag_2_O, MgO, and gradient HA (Fig. 3).

Bio-inert ceramics like zirconia and silicon dioxide have attracted much interest. Additionally, it has a high cell viability and strong load-bearing capability. Lee et al. [47] used the hydrothermal approach to create zirconia-coated porous Ti (Z-P-Ti), then used the sol-gel method to apply the coating. The investigated samples' highest cell viability and best load-bearing capacity were found in Z-P-Ti_55, composed of Ti samples with 55 wt% additions of NaCl (Fig. 4).

Z-P-Ti interacts with the surface of the cell in the way shown in Fig. 4. The strong adherence was achieved by Lubas et al. [52] by sandblasting an Al_2_O_3_ and ZrO_2_ sol-gel layer. In order to adsorb several complement proteins, Romero-Gavilan et al. developed a silica hybrid sol-gel coating (35M35G30T) on Ti.

### 4.2 Improving mechanical properties

#### 4.2.1 Enhanced wear resistance

Wear resistance can be considerably enhanced by depositing a thin layer of wear-resistant material onto the surface of a substrate using CVD. By modifying the surface properties of the substrate, the longevity of the substrate, as well as its resistance to wear and damage caused by friction, may be improved. According to T. Xue et al. [53], Surface modification technologies for biomaterials benefit greatly from Plasma Immersion Ion Implantation (PIII)'s ability to embed various components in the near-surface region of different substrates. One of the most significant advantages of PIII is that it allows for the efficient management of the modulation of the concentration and depth distribution of implanted ions in the substrate by modifying the implantation settings. Evidence suggests it can also improve biomaterials' bioactivity, antimicrobial characteristics, hardness, corrosion resistance, and wear resistance [54].

W Cui et al. [55] examined four coatings; two had TiN as their top layer, while the other had TiO_2_ as their top layer but with varying amounts of oxygen. TiN's multilayered film condition tribological performance is superior when sliding across bovine cortical bone. When comparing TiN and ZrN coatings, researchers found that partial substituting Ti atoms with Zr yielded superior fracture toughness and wear resistance. TiZrN-graded coating is suitable for application in artificial joints due to its resilience under stress and wear. Furthermore, Thangavel et al. [56] created Ag-doped NiTi (NiTi/Ag) coatings for pure Ti substrates. Human dermal fibroblast newborn cells demonstrated the greatest survival and best-developed actin filament network on the NiTi/Ag coating with 3 at% Ag.

The mechanical characteristics of titanium, the most widely used material for orthopedic purposes, may be diminished by sol-gel treatment, including its hardness and flexural modulus. D Horkavcova [57] discovered that 500 °C was the ideal annealing temperature for coatings to solve this issue, even though doing so lowered ductility. Coatings formulated from TiO_2_ boast several desirable physical properties, including great corrosion resistance, low friction coefficient, remarkable wear resistance, and high surface hardness. It was found that silver-containing titania was put on TiSi alloys [57] and commercially pure titanium (CP-Ti) by Yetim [58]. Orthopaedic applications are possible because of the materials' great corrosion resistance and low cytotoxicity.

Coatings are frequently utilized to improve implant surfaces' hardness, corrosion resistance, and mechanical qualities. Compared to Ti, surface coatings of Ti alloy are harder, more biologically compatible, more physically and chemically stable, and more resistant to corrosion in biological media [59]. TiN coatings can be deposited using several different processes, some of which have been documented in the literature: CVD [60], plasma ion implantation [61], and laser nitriding [62]. However, Ti6Al4V (Ti64) ion implanted layers have been shown in experiments to need to be removed after a few years [60]. Titanium nitride (TiN) coatings formed via CVD coating provide superior hardness characteristics, although they display limited adhesion properties. The outstanding performance of transition metal nitride coatings in thin-film resistors, tool coating, ornamental coating, and other areas has led to their increased attention in recent decades. These properties include protection against corrosion, high wear resistance, and low coefficient of friction. For the last 50 years, the TiN thin film coating has been used in various cutting tool applications because of its high hardness, low coefficient of friction, and corrosion resistance. The uses of TiN extended beyond the realm of machining and included plasmon-enhanced photocatalysis, thermophotovoltaics, photothermal tumor ablation, heat-assisted magnetic recording, and other fields. Numerous researchers have investigated the biomedical uses of titanium-based artificial implants because of their increased hardness, stronger adherence to the substrate, and increased resistance to corrosion and erosion [63].

### 4.3 Facilitating drug delivery

Researchers in the academic world keep looking for new ways to improve the delivery of medications in the hopes of improving therapeutic outcomes and reducing unwanted side effects. To deposit thin films or coatings onto different surfaces, the fields of materials science and nanotechnology frequently use the technique of chemical vapor deposition (CVD). Indirectly, CVD can improve drug delivery systems by allowing the development of specialized materials and architectures, even though it does not directly improve medication administration. The exact control over material properties made possible by chemical vapor deposition (CVD) allows for improved drug delivery systems and carriers in terms of efficacy, stability, and specificity [64]. For many pharmacological treatments, controlled drug delivery systems are beneficial because they allow for the targeted distribution of medication and the adjustment of delivery rates, which reduces harmful side effects and maximizes possible therapeutic value [65]. Designing a medication delivery system with these features is difficult while maintaining the medicine's therapeutic effects. Many drug delivery methods use a porous matrix functionalized with a polymer that responds to stimuli [66,67]. The stimulus-responsive polymer allows for the regulated diffusion of the medication in response to certain circumstances, such as Temperature or pH [68], and the porous matrix offers a large volume reservoir for the drug.

#### 4.3.1 Controlled release systems

Drug delivery systems that can be regulated and targeted have the potential to greatly improve medication treatments. They allow for precise drug administration and control the rate at which the drug is released. This capability may effectively minimize adverse side effects while concurrently maximizing the therapeutic efficacy of the treatment [69]. However, the responsibility of developing a drug delivery system with these characteristics while still preserving the therapeutic efficacy of the medication is a formidable challenge. Different drug delivery methods include using a porous matrix modified with a stimulus-responsive polymer. The drug is contained inside a substantial reservoir facilitated by the porous matrix, while the stimulus-responsive polymer controls the dispensation of the medication in reaction to variations in environmental variables, such as pH13 or temperature [70].

Because of the stimulus-responsive features of the polymer, the medication can be progressively delivered into precisely controlled cellular settings. In their study, Ram Prasad et al. [71] showed the use of porous silicon capped with polymeric components, whereby they developed hybrid hydrogels composed of poly(vinyl alcohol) and porous silicon (pSi). The purpose of these hydrogels was to facilitate theophylline's regulated release. Because the swelling capacity of PVA hydrogels is proportional to the amount of pSi particles cross-linked into the polymer network, changes to the release profile are possible. Mclnnes et al. [72] presented a polymeric drug delivery system sensitive to stimuli for combination chemotherapy. This approach enables the radiometric integration of two pharmaceutical substances into a uniform micro-composite composite of Hypromellose acetate succinate. Hypromellose acetate succinate exhibits insolubility in an acidic milieu but rapidly dissolves in a basic or alkaline milieu. The pH sensitivity of the composites exhibited a sluggish response, but their release kinetics were well controlled.

Thin films can now be uniformly coated on biodegradable pSi13 and other materials using an innovative chemical vapor deposition (CVD). Compared to wet chemical polymerization methods for functionalizing pSi drug delivery vehicles, iCVD's low temperature and solvent-free coating technique has clear advantages. The drug is protected from degradation or loss during the capping process by being preloaded into the pores before the surface is capped with the polymer. The efficiency and accuracy of drug loading are greatly improved as a result. The CVD method and its adaptability have been the subject of much research [73].

**Fig. 5:**
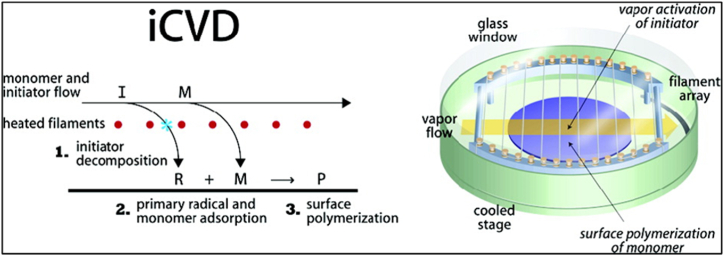
Initiated Chemical Vapor Deposition [73].

To perform the iCVD (Fig. 5), a specialized reactor (Sharon Vacuum) is utilized. Using a thermocouple, a nichrome (80 % Ni, 20 % Cr) filament excited to 285 °C. The deposition stage, kept at 20 °C using water cooling, was situated parallel to the heated filament and 2 cm above it. Improved parameters from the same publication were used to conduct an iCVD on (NIPAMco-DEGDVE) [73].

The primary comonomers used in this study were Aldrich 99 % N-isopropyl acrylamide (NIPAM) and Aldrich 99 % diethylene glycol divinyl ether (DEGDVE). The cross-linking agent used in this study was DEGDVE, whereas the radical initiator utilized was tert-butyl peroxide (TBPO), with a purity of 97 % obtained from Aldrich. To generate sufficient vapor flow, it was necessary to heat the NIPAM and DEGDVE to temperatures of 70 and 100 °C, respectively. However, the TBPO exhibited effective performance at ambient temperature. The flow rates of N-isopropyl acrylamide (NIPAM) (0.6 standard cubic centimeters per minute), diethylene glycol divinyl ether (DEGDVE) (0.1 standard cubic centimeters per minute), and tert-butyl per(oxy)benzoate (TBPO) (0.1 standard cubic centimeters per minute) were all regulated using mass flow controllers manufactured by MKS. Following the completion of the experimental protocol, the aggregate pressure inside the reactor was measured to be 0.5 Torr. A reference sample was prepared by applying a temperature-insensitive coating of poly (amino styrene) (pAS) using the initiated chemical vapor deposition (CVD) technique. The process of CVD has been used to characterize and showcase the application of this particular monomer. Laser interferometry was used to monitor the development of the film on an in-situ Si wafer during deposition. The deposition process was halted upon reaching a film thickness of 200 nm [73].

Atomic layer deposition (ALD) techniques allow for molecular or atomic-level control of ALD thickness via consecutive self-limiting surface reactions. To acquire thin films, the substrate undergoes a series of cycles whereby dosages of purging gas are introduced after two or more precursors. This process effectively eliminates any unreacted precursor molecules present in the gas phase. Most instances in which Molecular level deposition (MLD) occurs include the involvement of two chemical substances that undergo coupling activities. Due to their inherent self-limiting nature, these reactions proceed sequentially, occurring at the level of individual molecules, ultimately forming a film. ALD uses metalorganic precursors and oxidants such as oxygen plasma, water vapor, and ozone. The latter substance functions as an oxidizing agent, facilitating the decomposition of the organic matter present in the precursor. This process ultimately leads to forming an oxide layer devoid of oxygen. Achieving great conformality and satisfactory control over layer thickness at sub-nanometer scales is made possible by the self-limiting surface reactions of the MLD and ALD processes. However, this advantage comes at the cost of a relatively low deposition rate [74].

**Fig. 6:**
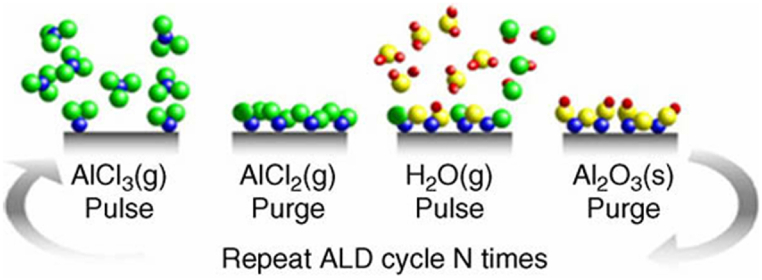
Atomic layer deposition (ALD) [75].

Fig. 6 shows the sequential procedure of ALD. With varying magnifications, three SEM images are shown. SEM Picture at 1 μm the microstructure seems more complicated than in the 5 μm scale since minute granules or particles are visible and the size appears more textured. Structures resembling needles are seen at 400 nm scale, suggesting a particular microstructural deposition. The structure becomes more distinct, revealing their intricate morphology and maybe even their orientation or arrangement.

#### 4.3.2 Localized drug delivery

Several obstacles arise in the process of creating biomaterials for medication delivery. The key considerations for implantable drug delivery systems include enhancing biocompatibility, minimizing dimensional drawbacks, enabling adjustable release rates, reducing nonspecific elution, preventing excessive release or "burst" release, ensuring scalability of material production, and evaluating the post-drug release effects [76]. Concentrating drug delivery to a specific region will decrease systemic drug concentrations.

This strategy can reduce or even eliminate the need for many future therapies when combined with keeping medications active while in confinement. Targeted delivery of chemotherapeutic medications is an example of this, as it has been shown to reduce systemic adverse responses while simultaneously increasing the treatments' therapeutic efficacy. Regulatory agencies have authorized a number of drug delivery systems that are now being used in clinical trials. These systems include transdermal drug delivery patches, polymer-drug conjugates, local chemotherapy, controlled administration of cancer therapies, and liposomal systems [77].

Modifying diffusion-based systems and utilizing microchip deliveries are two examples of long-term delivery strategies with proven track records. R Lam et al. discussed using a few basic tactics based on Nano-diamonds (NDs) to achieve sustained, regulated drug delivery over an extended period [78]. Definitionally, NDs are a platform for several biological uses, the most prominent of which being cancer treatment. These applications range from primary components inside polymer hybrid microfilms to systemic modalities.

Hence, the circumvention of drug penetration issues can be achieved through the implementation of permanent and localized release mechanisms. In spheroid research, continuous infusion of nano formulations prolongs drug circulation time (DOX), resulting in a higher compound concentration in cells closer to the spheroids' periphery. These results suggest that the therapeutic agent is retained and distributed more evenly across the tumor. In essence, by using long-term procedures that progressively remove successive layers of cells, it is possible to target malignant cells experiencing diminished food and energy supply and impeded response to therapeutic therapies. Using this method, cells that were previously inaccessible can now be reached. New insights into drug penetration may be gained using cutting-edge in vitro and in vivo models [79].

**Fig. 7:**
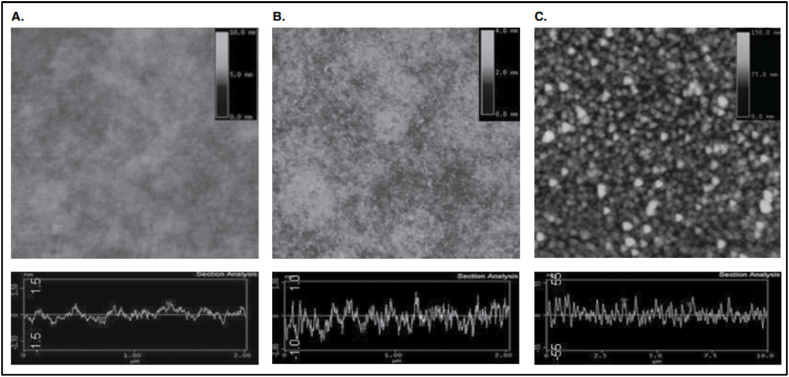
AFM scans of successive layers of glass (A), poly-l-lysine (B), and ND (C) during the process of depositing successive layers [81].

According to IF Tannock [80], slowing drug penetration has been demonstrated to reduce chemotherapy's effectiveness in multicellular layer models, particularly in cells furthest from blood vessels. Removing progressively deeper layers of cells throughout treatment is a major consideration, especially at the periphery and near blood arteries. Malignant cells are more difficult to treat since they receive less nutrients and energy due to a slow, methodical approach that removes successive layers of cells. New, cutting-edge in vitro and in vivo models would be helpful for drug penetration research. A localized drug delivery method that permits prolonged dose control and provides a firm basis (Fig. 7) for greater biocompatibility and flexibility would be superior to the current technology, which has these undesirable side effects.

The device was initially designed as an adjunctive treatment for early-stage implantation after tumor removal to use its strong and prolonged drug-release capabilities and targeted chemotherapeutic effectiveness to decrease or potentially eliminate the need for further radiation therapy. If this works, much less medicine will be utilized, resulting in better patient care, fewer adverse effects, and no need for additional procedures. Modifying NDs conjugation with disease-specific agents expands the device's potential uses beyond oncological treatment; it could be used for anti-inflammatory therapies or any other purpose that requires precise local application to a disease site [81].

### 4.4 Preventing corrosion and oxidation

CVD coating deposits tiny layers of material on surfaces. These coatings can protect against oxidation and corrosion and can be tailored. CVD coatings protect substrates from the environment. These thin coatings protect substrates against oxygen, moisture, and chemicals. CVD carefully controls coating thickness. More oxidation and corrosion protection come from thicker coatings. Some chemically inert CVD coatings are best. They can be employed in tough or corrosive settings because they don't react with air's natural chemicals or gasses. The intended usage and expected operating conditions must be considered while choosing coating materials and production parameters. Due to their adaptability and modification ability, CVD coatings defend against corrosion and oxidation in aerospace, automotive, electronics, and other industries. The analysis of the technology and properties of materials used for gas turbine and aircraft engine parts reveals notable limitations [82].

#### 4.4.1 Biomedical implants in harsh environments

Carbon-based materials have become superior to biomedical materials due to their superior mechanical capabilities, reduced friction on the surface, wear and tear, and corrosion resilience. Carbon-based coatings for biomedical implants have shown encouraging outcomes in experimental trials. Their usefulness stems from their capacity to quell the thrombo-inflammatory responses triggered by the immune system in response to the implantation of a foreign body.

Diamond-like graphene, silicon carbide, and pyrolytic carbon are carbon coatings studied and used in biomedicine. Their unique atomic structure and organization make them useful in many disciplines. Carbon-based precursors are distinctive and hard. Thus, they can only be employed with a particular coating method for manufacturing nanostructure. This work explores several coating strategies based on the substrate material, implant type, and coating layer thickness. This decreases the coating material for large substrates. Coatings can improve wettability, hydrophobicity, corrosion resistance, surface hardness, texture, heat, and electricity insulation. Corrosion resistance is comparable between additively made and traditionally made NiTi alloys in these components. Here, the additive manufacturing process won't reduce the NiTi components' corrosion resistance. The XRD and SEM analysis of the corroded NiTi sample indicates the formation of biocompatible corrosion products, which could benefit bone implant application [83]. Despite the benefits noted above, coating procedures have inherent limitations that reduce reliability. The primary considerations revolve around the properties of the coating materials, including their melting point, availability in various forms (foils, powders, rods), and biocompatibility.

Additionally, it is crucial to account for the adverse thermal effects, such as distortion, cracking, and delamination, as well as the detrimental consequences of inadequate atmospheric protection, such as the infiltration of inclusions and contaminants into the substrate [84]. The semiconductor industry makes extensive use of the CVD method, which involves a high vacuum and produces a coating layer that is solid, of high quality, and highly resistant to any substrate [85]. CVD protects constant-contact moving parts from corrosion and wear. Heat elements heat the substrate to enhance chemical interaction with the evaporated CVD materials pushed in from the right. CVD may produce graphene, polymers, diamond, fluorocarbons, oxynitrides, nitrides, carbides, graphene, polymers, diamond, fluorocarbons, nanotubes/fibers/nanofibers, W and Ti. These materials come in microstructures of amorphous, monocrystalline, and polycrystalline [85]. Since only the heated area may be coated, material waste is reduced using CVD. To further this capacity, lasers controlled by a computer might be used to heat only the desired parts [85].

#### 4.4.2 Protection of implant materials

Many coating methods and materials are available to safeguard part of a structure or framework against mechanical or chemical damage. This protective function helps keep production costs down by preventing the need for replacement components. Tailored plastic materials, polymers, bio-glasses, ceramics, and metallic alloys are just some of the coating materials available, allowing designers a wide range of options for providing long-lasting safeguards [85].

Various industries' uses and requirements necessitate a wide range of coating techniques. Depending on the specifics of the process, the microstructure, efficacy, appropriateness, and durability of the resulting material can vary greatly. However, coating techniques are most beneficial when applied when particular functions, such as corrosion and wear prevention, are critical [86].

Since the materials used for a coating are what the coating will ultimately protect, they are the most crucial aspect of the coating's performance. Many different materials can be used to create a shield. Among these are metals, ceramics, and plastics. The ideal composition of the deposited layer might be challenging to determine due to the vast variation in coating processes and material characteristics. While looking for promising new possibilities is important, researchers should focus on the industry standard solutions first. The feedstock materials exhibit diverse melting temperatures, mechanical characteristics, and chemical properties. However, their common attribute is their ability to resist corrosion or wear. In addition to these considerations, the fact that materials can be found in various forms (powders, rods, plates, and wires) makes it difficult to find a universal solution. While other methods are available for protection, including heat treatment, mechanical treatment, mechanical/chemical finishing, and polishing, this evaluation primarily concentrates on the predominant coating techniques, materials, and their surface modification quality [87].

Vaporization-based methods are often used to create a coating layer, which is widely applied in tool coating and safeguarding machine sliding components. This is primarily due to the thin film nature of the coating and its remarkable resistance to corrosion and wear. These coatings provide a substantial, cohesive layer that exhibits strong adhesion to the underlying material. Micro-Arc Oxidation (MAO), a high-voltage variant of the anodization process, finds widespread use in the treatment of biomaterials utilized in bone implants and several other medical devices [85]. Drug-loaded hydrogels can be administered less invasively because of their malleability and capacity to build tissue constructions in situ. The in situ formability of injectable hydrogels with biodegradability enables the efficient and uniform encapsulation of medications and cells in both laboratory and living organism settings. This facilitates a less invasive surgical procedure in living organisms, resulting in smaller scars and less patient discomfort [88].

## 5 Illustrative examples of CVD coatings for biomedical implants

Chemical Vapor Deposition (CVD) coatings find significant utility within biomedical implants. Using these coatings makes it possible to augment the performance and biocompatibility of implant materials, rendering them more appropriate for utilization within the human body. The prevalent applications of CVD coatings in the domain of biomedical implants are as follows.

### 5.1 Orthopedic implants

Orthopedic implants that integrate with the patient's bone naturally produce the best long-term benefits. We highlight the possible advantages of such coatings in the context of orthopedic implants, including improved integration with the surrounding bone, improved mechanical characteristics, facilitated surface chemical functionalization, and anti-microbial properties. CVD has become the most used method for depositing diamonds as a surface coating because of its efficiency, precision, capacity to cover enormous areas, and scope for diamond development on substrates. CVD creates a plasma below the surface using carbon radicals and dissociated hydrogen using either heated filaments or microwaves. Cobalt-chromium (Co-Cr) alloys are often used for commercial orthopedic implants because of their notable resistance to corrosion and abrasion, favorable biochemical characteristics, and moderate strength [89].

However, Metal ions released from the alloys into the body fluids (serum, urine, etc.) can accumulate between the implant and the tissues, causing an inflammatory reaction [88]. This can happen if the passive film between the implant and the tissues breaks down over time due to long-term fatigue friction and wear of orthopedic implants. Cutaneous allergic responses, osteolysis, clinical implant failure, and distant site accumulations are some of the issues that may arise from the high concentration of metal ions and wear debris at the implant-tissue interface.

**Fig. 8:**
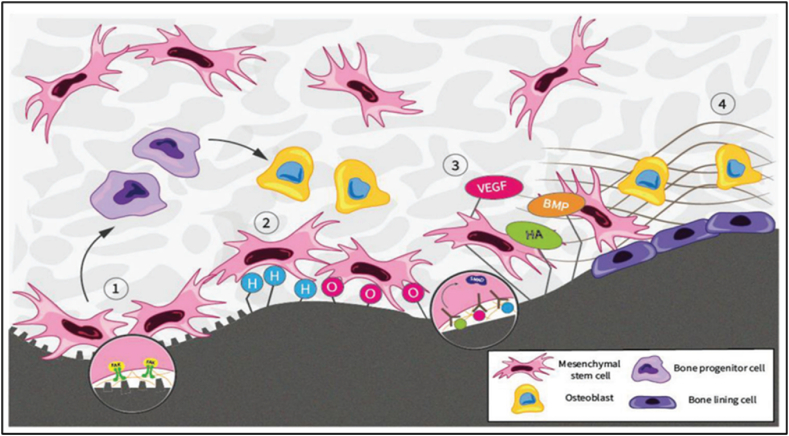
MSC at the diamond-bone interface [90].

However, limited research has been done on CVD TiN coating on the Co-Cr alloy used in orthopedic implants. To better drive Mesenchymal Stem Cells (MSC) toward osteogenesis and improved osseointegration, Fong et al. investigated and analyzed the potential of diamond and its primary qualities. MSCs, or mesenchymal stem cells, reside in the dynamic and complex niche. MSCs keep their unique abilities of differentiation, proliferation, self-renewal, and quiescence inside this niche. Since diamonds' physical and chemical properties provide informative stimuli that directly affect cell behavior and the fate of stem cells, we also perform a more in-depth study of these factors (Fig. 8) [90].

The present discourse aims to elucidate the many roles and interactions involved in bone regeneration, explicitly focusing on four key aspects. Surface roughness serves as a means of presenting osteoinductive signals to mesenchymal stem cells (MSCs), hence facilitating the differentiation of these cells into osteogenic progenitor cells and osteoblasts. The impact of O- and H-terminated diamond surfaces on the recruitment, proliferation and adhesion of mesenchymal stem cells (MSCs) at peri-implant sites before osteogenesis is influenced by surface chemistry. Diamonds' surface topography and mechanical stability contribute to bone mineralization, apposition, and long-term implant fixation (Fig. 8) [90].

### 5.2 Dental implants

Bone structure is preserved and strengthened with dental implants. This means they do much more than fill in a gap; they also safeguard natural teeth by maintaining bone density. The moist penetration division of dental implants is visible in the oral cavity. Dental calculus and plaque attach to implant surfaces more readily than natural teeth. They can only be removed via a scaling procedure, which is a crucial step in the healing process for implant maintenance over the long term. The presence of carbide coating on Ti greatly reduced the indentation depth and breadth compared to pure Ti, as seen by the results of the Martens scratch test and the ultrasonic scaler abrasion test, confirming its high abrasion resistance. These findings demonstrated that surface carbide coatings might be a useful way to enhance the wear characteristics of Ti implant components that need strong abrasion resistance, such as abutments for dental implants [91].

Titanium carbide (TiC) has exceptional properties that make it suitable for various industrial applications. These properties include an exceptionally high level of hardness, an elevated melting temperature, and remarkable thermal and chemical stabilities. High-temperature structural modules in engines and heat exchangers and wear-and abrasion-resistant components like forming dies and cutting tools are all made of ceramics based on titanium. Comparing titanium matrix composites (TMCs) to steel and nickel-based materials, TMCs exhibit higher specific strength and stiffness [92].

**Fig. 9:**
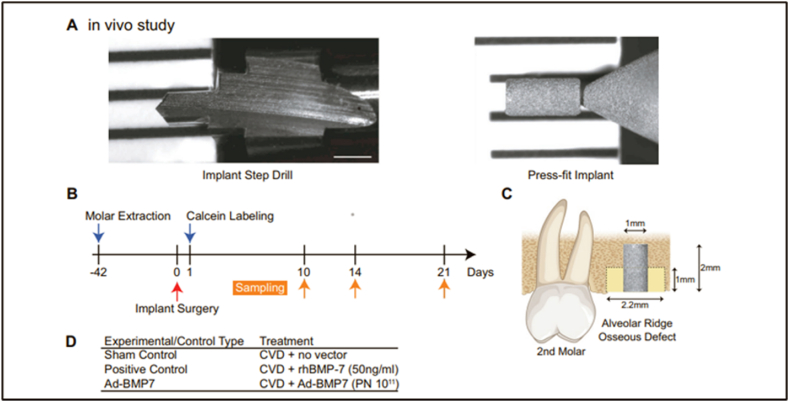
Design of the in vivo study's experimental setup [93].

Maekawa et al. [93] used Sprague-Dawley rats as subjects in their in vivo study to examine the effects of cardiovascular disease using CVD coating and gene delivery on titanium implants. Improving Bone-to-Implant Contact (BIC) and encouraging bone regeneration were the key aims of the study. Gene-encoding vectors can be anchored onto biomaterial surfaces using functionalized para(cyclo)phanes, and local delivery of a vector expressing Bone Morphogenetic Protein (BMP) can improve alveolar bone volume and density in vivo. This work aims to enhance bone regeneration and bone-to-implant contact in vivo by combining the CVD technology with BMP gene delivery on titanium. Fig. 9 illustrates the integration process. Uniform bone defects were generated to establish a standardized implant location. Fig. 9(A) displays a custom-made step drill on the left and a surface-treated titanium dental implant on the right. The scale bar indicates a measurement of 1 mm. The experimental schedule of the in vivo research included the following procedures: implant implantation, extraction of the first molars on both sides, and subsequent evaluation of results using micro-CT and histological examination. Fig. 9(C) presents a schematic representation of inserting a titanium implant into the alveolar bone and the corresponding percentages of bony defects created by the surgical procedure. The table presents the experimental and control treatment groups. Following the implantation of 108 subjects, 54 rats were assigned to the study. Twelve split-mouth implants were allocated to each group, with the surgeon's identity concealed. The in vitro enhancement of osteoblast development and osteogenic potential is achieved using chemical vapor deposition, specifically by introducing a BMP-7 gene-expressing adenovirus onto titanium surfaces. Potentially improving alveolar bone regeneration and increasing the proportion of bone-to-implant contact (BIC)-like results, large doses of locally given rhBMP-7 in vivo [93].

### 5.3 Cardiovascular implants

**Fig. 10:**
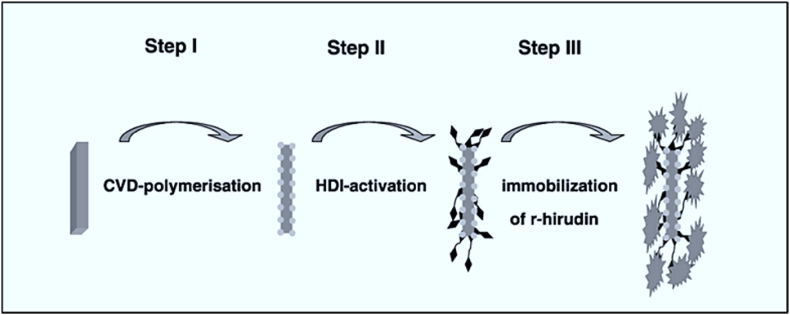
Strategy for surface biomimetics to enhance biocompatibility (HDI: hexamethylene diisocyanate) [94].

One potential solution to address the current challenges associated with metal stent complications is the development of a biocompatible covering that emulates the properties of a metal surface while actively inhibiting the pathways that contribute to restenosis. The functionalized paracyclophanes were CVD polymerized to coat nitinol coronary stents. The monomers 4-amino paracyclophane, 4-hydroxy methyl paracyclophane, and paracyclophane-4,5,12,13-tetracarboxylic acid dianhydride have all been effectively synthesized in earlier investigations. The polymerization approaches were implemented within an appropriate experimental configuration for the chemical vapor deposition (CVD) polymerization process, as seen in Fig. 10 [94].

Using these polymer coatings as interfaces provides functional groups for conjugating biomolecules, eliminating the need for further modification procedures. The successful immobilization of recombinant hirudin has provided evidence for the feasibility of this concept. The blood compatibility of bio-actively coated metallic coronary implants has been improved by surface-bound r-hirudin, as shown by lower thrombogenicity and significantly reduced platelet adhesion seen in vitro [94].

### 5.4 Neural implants

The coating of implantable medical devices is one area where the success of biomaterial development has proven vital. Insulating coatings on pacemaker leads offer similar protection for the implanted device. The biocompatibility of implants can be improved with the help of medical device coatings [95].

Kozai et al. [96] wanted to create and evaluate an ultra-small organic electrical microelectrode with a subcellular cross-sectional dimension. We aim to develop a flexible electrode with enhanced strength and enough electrical properties for neural recording. Additionally, we seek to incorporate improved bioactive features that enable the regulation of intrinsic biological processes. Composite materials were created to expand the possibilities of microelectrode design to meet specific functional needs for long-term performance. Previously, these criteria were limited by the inherent constraints in strength, size, and electrical trade-offs often associated with traditional technologies.

**Fig. 11:**
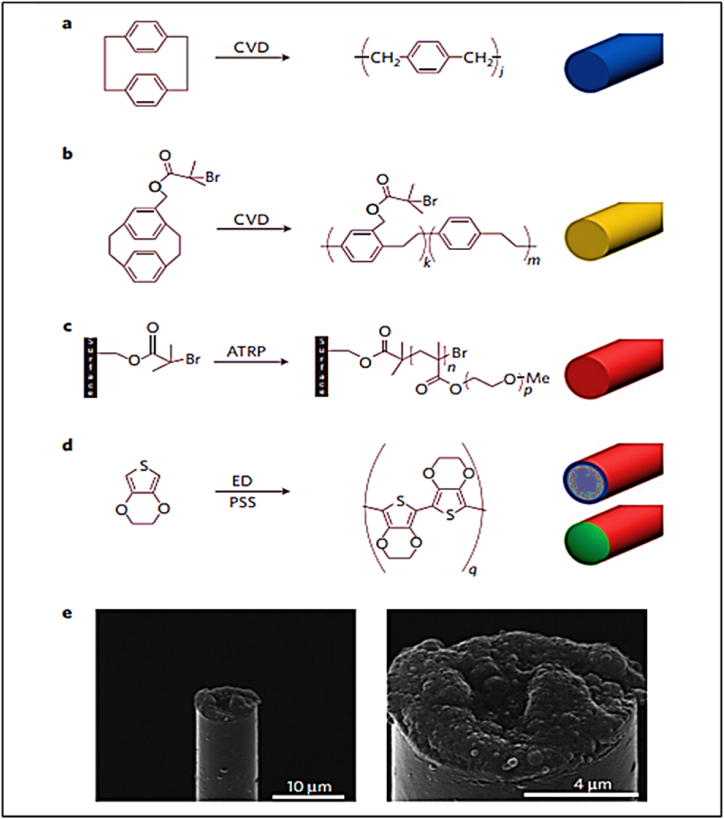
Micro thread electrodes.(a–d) Preparation of an MTE; e) SEM images of a fully assembled, functional MTE [96].

The Micro Thread Electrodes (MTEs) were manufactured by affixing carbon fibers with a diameter of 7 μm onto a printed circuit board optimized for microelectrodes. Subsequently, carbon fiber was subjected to CVD polymerization, resulting in the deposition of a poly(p-xylylene) layer with a thickness of 800 nm (Fig. 11(a)). The selection of poly(p-xylylene), often known as parylene-N, was based on its exceptional characteristics, which include a low dissipation factor, high dielectric solid strength, and a consistent dielectric constant that is unaltered by variations in frequency. A layer of the functionalized polymer poly((p-xylylene-4-methyl-2-bromoisobutyrate)-co-(p-xylylene)) with a thickness of 50 nm was applied onto the fiber coated with poly(p-xylylene) using the CVD polymerization technique (Fig. 11(b)). This particular polymer serves as an initiator group for future Atom Transfer Radical Polymerization (ATRP) processes. A carbon recording site is exposed at the tip by cutting away the insulation (Fig. 11(c)), and the recording site is coated with PEDOT by electrochemical deposition (Fig. 11(d)). SEM images of a fully assembled, functional MTE (Fig. 11(e)).

The predominant focus of scholarly investigations revolves around compounds closely associated with graphene, namely graphene-oxide (GO) and reduced-graphene oxide (rGO) elements. Nevertheless, incorporating graphene oxide (GO) with other electrical conductivity materials, such as conductive polymers and metals, becomes imperative to construct electrodes. The extensive surface area of rGO contributes to reduced impedance and increased charge-injection capacity (CIC), crucial factors in brain recording and stimulation [97].

### 5.5 Drug-eluting implants

Polymeric films often cover medical implants to improve biocompatibility and provide a long-lasting matrix for controlled medicine release. Drug-eluting matrices are often separated from the medical implant by a priming layer in these coatings.

The CVD technique deposits this family of coatings onto a surface, calling for high temperatures, low pressures, and sophisticated machinery [98]. One effective method for coating metallic implants involves electro-polymerizing conducting polymers onto stainless steel [99]. Distinctive coatings were created by modifying the deposition solution's monomer ratio, electrochemical conditions, and chemical structure. The non-toxic and non-biodegradable properties of the coatings were verified by High-Performance Liquid Chromatography (HPLC) and gel permeation chromatography testing. In addition, there were no noticeable changes in the surface structure over eight months of exposure to a physiological solution [100].

**Fig. 12:**
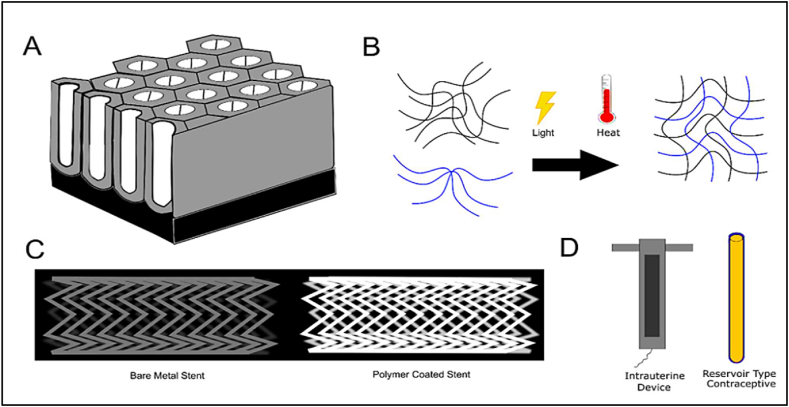
Illustrations from several IDDSs**,** A) The payload to be delivered can be inserted within hollow titanium nanotube arrays. B) External triggers like light and temperature can start polymerization in situ-formed implants such as hydrogels. C) One advantage of polymer-coated drug-eluting stents over bare metal cardiovascular stents is the ability to deliver antiproliferative medicines, which reduces the incidence of in-stent restenosis. D) Implantable reservoir-based contraceptive implants offer a cost-effective and minimally invasive option compared to Intrauterine Devices. (IUDs) [101].

A combination of aspirin and atorvastatin was dip-coated onto a 3D-printed porous Polycaprolactone (PCL) vascular stent by JC Quarterman et al. [101], reducing blood levels of low-density lipoprotein cholesterol and restenosis the constriction of blood vessels would be the system's practical use that is described in Fig. 12. They verified the coating's deposition and surface characteristics using X-ray photoelectron spectroscopy and Fourier transform infrared technology. Fig. 12 depicts representative illustrations from several Infectious Disease Detection and Surveillance (IDDSs).

## 6 Biocompatibility and safety considerations

Biocompatibility and safety regarding CVD coatings' potential use in medical and biological settings must be arranged. To guarantee the safety and effectiveness of cardiovascular disease (CVD)-coated devices while in contact with humans, it is essential to undertake a comprehensive and rigorous process of material selection, testing, and compliance with regulatory standards. Conducting a thorough assessment and testing process is vital to establish whether CVD-coated materials meet the required safety criteria for their intended application in medical or biological contexts.

### 6.1 Ensuring implant biocompatibility

The need for in vivo biocompatibility is arguably the most crucial quality of a biomaterial. Although the phrase "biocompatibility" lacks a consensus definition, "the ability of a material to perform with an appropriate host response in a specific application" [102] comes close to capturing the concept. The specific parameters essential to allow osseointegration have yet to be established despite identifying several key factors [103].

**Fig. 13:**
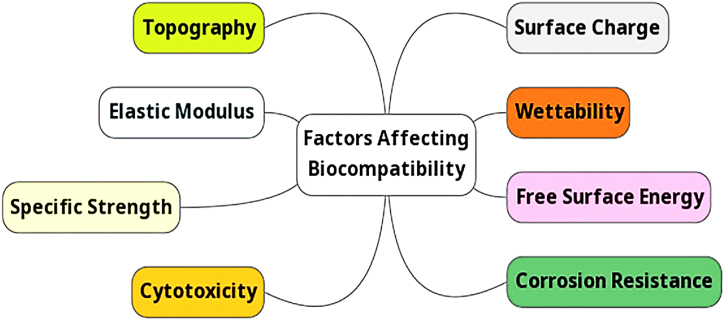
Factors affecting the biocompatibility [104].

Fig. 13 shows the factors that affect the biocompatibility. The ability of metallic biomaterials to withstand corrosion is a crucial need. The body's biological circumstances, consisting of interstitial fluid containing electrolytes (such as Na+, Cl, K+, PO4-)plasma proteins, and other substances that may come into contact with the surface of biomaterials, will be exposed to these materials. Surface charge significantly impacts biocompatibility, although the exact mechanism is unclear [104].

A biomaterial's surface charge significantly impacts the first plasma protein binding upon implantation. Compounds with the potential to be cytotoxic or genotoxic should not be present in the perfect biomaterial. Furthermore, they shouldn't cause the body to have a prolonged hypersensitive reaction. The biomaterial's topography will influence osseointegration; creative techniques have been devised to alter the titanium surface to have desired topographical characteristics without compromising titanium's overall characteristics. The resistance of a substance to deformation under stress is known as its elastic modulus. Titanium and its alloys have lately been recognized as promising biomaterials for orthopedics use. When orthopedic implants are inserted, prosthetic joint infection and aseptic loosening are prevalent issues. Developing orthopedic materials with appropriate qualities at the biomaterial-tissue interface has focused heavily on surface modification innovations to ensure implanted devices' long-term viability [104].

However, the impact of the principal underlying surface charge on biocompatibility is not yet known. The surface charge of the biomaterial significantly influences the phenomenon of initial plasma protein binding during implantation. This procedure results in the creating of a protein conditioning film, which can efficiently regulate the charge of the material surface. Therefore, this charge modulation is essential in regulating the adhesion of both bacteria and mammalian cells. The surface-bound host factors potentially possess distinct receptors that may interact with tissue cells and colonizing microbes. For example, titanium's positive charge attracts albumin and fibronectin, two proteins with negative charges, leading to initial noncovalent interactions [105]. Bacterial adherence to biomaterial surfaces is promoted by fibronectin, but albumin and whole serum appear to hinder it [106]. Hematological, cardiac morbidities, vertigo, deafness problems (polycythemia), and hypothyroidism are just some of the biocompatibility concerns brought up by cobalt use [107]. Wearing down the material can cause lasting changes in the metal composition of the blood and higher levels of metallic components in various tissues, such as the kidney and liver. This applies to both stainless steel and cobalt-based alloys [108].

According to Nakai et al. [109], The precursor temperature employed in CVD can affect the final coating's shape and thickness. Because of this, CVD can be used in a wide variety of industrial contexts. The issue of delamination, which is similar to PVD in practice, complicates CVD surface modification of materials. This factor is significant when this technology is used in orthopedic devices. Giavaresi et al. [110] found that coating titanium implants with TiO_2_ via chemical CVD was successful. This coating facilitated osseointegration in the femoral bone of rabbits at both the 4-week and 12-week time points. Various antimicrobial coatings, including poly(diethylaminomethyl styrene, have been applied using CVD techniques. Martin et al. [111] found that using this method significantly reduced viable Escherichia coli bacteria by a factor of 4 log. A thin gel coating forms on the surface when a specimen is submerged in a sol, a colloidal solution, and then subjected to polycondensation. The extra moisture is then removed using a drying process. Because of its proven effectiveness in efficiently coating complex structures like orthopedic implants, the method described in the literature sees wide application [112].

Furthermore, this approach exhibits a cost-effective nature and necessitates moderate processing temperatures, hence facilitating the preservation of the material's overall mechanical characteristics [113]. Using the sol-gel technique, a porous coating comprising silver nanoparticles has been successfully deposited onto titanium substrates. This intervention has been proven to significantly decrease S. aureus adhesion by an impressive 99.3 percent within 24 h. Sol-gel MgO films produced on titanium substrates are biocompatible with osteoblast cells in vitro, as shown in a work by Shunzhi et al. [114]. There was also a little antibacterial effect against *E. coli* seen with these films.

### 6.2 Addressing potential toxicity and allergic reactions

Careful material selection, extensive testing, quality control procedures, and adherence to regulatory norms are all necessary to address the potential toxicity and allergic reactions related to CVD coatings. The safety of CVD-coated products used in medical and biological applications can be improved by coordinated efforts involving specialists in materials science, biocompatibility testing, and regulatory affairs.

Research by Chen et al. [115] on magnetron-sputtered and thermally oxidized Ta-doped Ti-O coatings showed comparable outcomes. Adding nitrogen to TiO_2_ (optimal at TiN0.4O1.6) has been shown to lessen thrombocyte adhesion and fibrinogen adsorption [116]. In vitro studies with osteoblasts, fibroblasts, and epithelial cells showed that titanium oxide multi-component coatings doped and alloyed with N, C, Si, Zr, and Ca elicited no inflammatory reactions or cytotoxic effects, making them well-suited for osseointegration [117]. The term "cytotoxicity" describes the potential for a drug to harm cells. Cell death, malfunction, DNA damage, membrane rupture, and inflammatory responses are all components.

All films corroborated fast cell differentiation and high rates of osteoblast proliferation. Compared to control substrates, cell growth was promoted on Ca- and P-doped TiOx films. Cells in the calvaria of developing rats showed high bioactivity by secreting bone matrix proteins and depositing minerals into the collagenous matrix. In experiments using rat calvaria and hip defect models, preliminary indications of bone growth were seen on titanium substrates coated with these materials [118]. The films had considerably more growth and survival than the control substrates. The in vitro generation of cytokines and the adhesion of transplanted endothelial cells were investigated by Lehle et al. [119] using polymers coated with PE-CVD titanium carboxy-nitride. Human saphenous vein endothelial cells appeared unaffected. Additionally, there was a small decrease in cytokine production and no noticeable change in the expression of baseline adhesion molecules.

Hydrogenated carbon films containing titanium were used in bone marrow cell culture research by Schroeder et al. [120]. The production of these films was achieved by integrating magnetron sputtering and Radio Frequency Plasma-Enhanced Chemical Vapor Deposition (RF PE-CVD) techniques. The researchers found that bone cell proliferation was significantly enhanced, whereas osteoclast activity was significantly reduced in these films. The high bioactivity exposes Humans to increased oxidative, cellular, and genotoxic stress. The magnitude of the impacts may be proportional to the size of the diamond surfaces. However, other authors showed that Diamond powder particles were bioinert. Superoxide and lipids from the cell membrane are two byproducts of hemolysis. This infectious process occurs within the human body and responds well to treatment when illness is present. The drugs used by doctors to treat this sickness are only partially effective because their mechanism of action is limited to symptomatic alleviation. Diamond powder particles show promise as a potential therapeutic method for treating hemolytic anemia [121].

### 6.3 Regulatory compliance and ethical considerations

A patient's ability to follow post-operative instructions and guidelines supplied by the medical team is crucial to ensuring a positive outcome and reducing the risk of complications following the implantation of a medical device. Only when patients follow post-operative care instructions will biomedical implants function as intended; providers should give patients thorough, correct instructions and everything they need to complete their treatment. Ethical considerations should be given substantial weight at every stage of developing and implementing biomedical implants. Ethical concerns related to a topic may change throughout time as a result of developments in technology and shifts in cultural norms. Healthcare practitioners, researchers, and policymakers must maintain steadfast vigilance to handle the ethical concerns provided by biomedical implants effectively. Protecting individual rights and interests while maximizing the benefits to patients and society is crucial. Flexible, soft materials can reduce tension in brain tissue caused by micromotion. When exposed to tissue, comprehension of the material's behavior is paramount in advancing brain implant designs with enhanced efficacy. Intracortical microelectrodes are often used to capture the electrical signals emanating from individual neurons or groups of neurons [122]. Neuroscientists study neural connections and the development of neurodegenerative illnesses by implanting microelectrodes into animal models of health and disease [123]. Integrating biotic and abiotic components in brain-computer interfaces used in rehabilitation may be achieved via microelectrodes [124]. The capacity to reliably capture high-quality signals over time is currently a limitation of intracortical microelectrode technology [125].

Intracortical microelectrodes are prone to material, mechanical, and biological failure modes, and it is increasingly believed that neuroinflammatory processes play a key role in triggering these failures. Multiple factors have prompted researchers to hypothesize that microglia and infiltrating blood-derived macrophages play a major role in modulating neuroinflammatory processes after microelectrode implantation [126]. Popular theories also include mechanical mismatch in implanted device regions [127]. According to studies [128], micromotion and tethering forces can cause harm to brain tissue near implants. For instance, arterial pulsation can cause a displacement of 2–4 μm in cortical tissue in the rat brain, while breathing can cause a displacement of 10–30 μm [129]. Injuries to the endothelium barrier or cortical tissue may spread and worsen the foreign body response to microelectrodes, hindering wound healing processes [130].

**Fig. 14:**
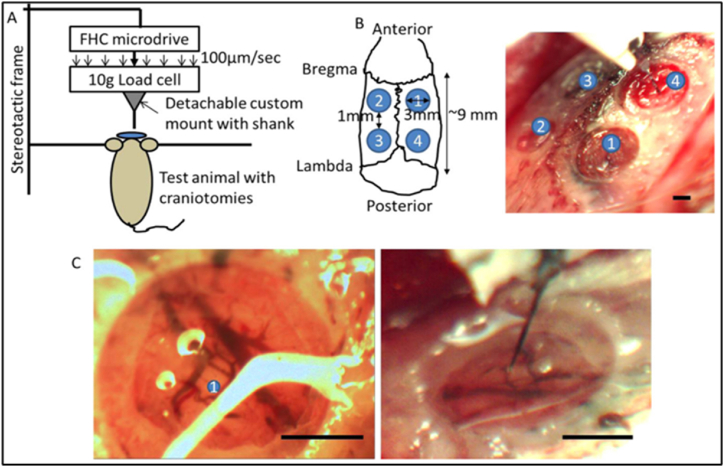
(A) illustration of the experimental setup used to measure force-displacement curves in brain tissue in vivo [131].

The experimental setup to measure force-displacement curves in brain tissue in vivo is illustrated in Fig. 14. There is a way to set up an experiment to quantify force-displacement curves in real time in living brain tissue. A load cell (10g, Futek, Irvine, CA) is attached to the stereotactic frame in a direction perpendicular to the craniotomy once the animal is securely fixed to it. A nanocomposite shank with a load cell is lowered into the craniotomy at a pace of 100 m s1 using an FHC microdrive (Bowdoin, ME) that can handle bare silicon, PVAc-coated silicon, or nanocomposite materials. Fig. 14 (A) Four craniotomies have been roughly shown in the animal's skull. For the force measurements, gel foam was moistened with 0.9 % saline and placed over the craniotomies. In this example photo, a PVAc-coated silicon shank is implanted at 1 mm depth in craniotomy [131].

The images on the left and right show the same craniotomy with the dura intact before (left) and after (right) reflection. The location of minimum superficial vasculature in the brain is marked, and a bare silicon probe shank is put there to record force. Note that the silicon shank is at a right angle (90°) to the skull. The camera angle is to blame for the skewed perspective. In both Fig. 14 (B) and (C), 1 mm is shown by the scale bar [131].

Many research groups, including the Capadona group, have shown that using compliant implants minimizes neuroinflammatory responses [132]. Stresses in live brain tissue are thought to cause strain and micromotion; however, experimentally, it has not yet been proven whether or not compliant microelectrodes mitigate these effects. The dynamic changes in the material properties of brain tissue are said to be mirrored in the mechanical stresses experienced by the tissue owing to micromotion.

## 7 Limitations of CVD coating in biomedical implants

High temperatures are frequently needed for traditional CVD techniques, which may harm the structural integrity of some biomedical implants, particularly those composed of polymers or other materials that are sensitive to temperature. The high coating temperature and certain corrosive gas byproducts generated during the coating process may result in an unwanted reaction at the implant–coating interface, contingent upon the chemical composition and reactivity of the implant substrate [133]. Insufficient biocompatibility, a higher rate of deterioration (for magnesium-based alloys), an inflammatory reaction, infections, inertness (for silver, titanium, and co-cr alloys), a mismatch in shielding stress, elastic modulus, and excessive wear can all contribute to the failure of metallic implants during surgical procedures. CVD restricts the choice of materials for both the coating and the substrate, potentially limiting the application of CVD in creating implants that require specific material properties [134].

Although CVD can produce extremely pure materials, the reaction chamber and precursor gases can potentially contaminate the process processing, and enhance the "mean free path" for atoms and high-energy ions to collide, thereby reducing gaseous contamination to an acceptable level. The biocompatibility of the implant, which is essential in medical applications, might be jeopardized by any infection. Since the materials used to make these implants are diverse and include metallic materials like titanium, cobalt chromium, and its alloys, composites, bio-ceramics, and polymers that come into constant contact with bodily fluids that can be corrosive, they frequently fail and eventually fracture. The way different implants corrode and the impact of the surface oxide layer and corrosion products on implant failure [135].

According to A Barik et al. [136], a comprehensive summary of various titanium implant forms focuses on the advancements and modifications made in drug delivery methods in recent years. A peri-implant infection and osseointegration failure are among the most challenging post-implantation scenarios that might arise. The durability and efficacy of implants, whether given topically or intravenously, are greatly influenced by biologics and pharmacological molecules. The development of a trustworthy biologic covering for orthopedic implants is being advanced thanks to the encouraging outcomes of the continuing research into these areas. This study considers and analyses several variables: kind, carrier, technique, thickness, stability, and in vivo degradation. The coating degradation and the movement of the drug molecules in the reservoir would follow the predicted mathematical relationship. Improving industrial output necessitates the creation of a practical commercial method [136].

Whether the implant will be successful or not depends on the coating materials on the implant, its appropriate design, and biocompatibility. Due to the complexity of the CVD process, it is necessary to have advanced equipment and to exercise rigorous control over the process parameters. It is a commonly held belief that for implanted materials to be successful in clinical settings, they must be compatible with the mechanical characteristics of natural tissue and build a stable interface with the tissue surrounding them [137]. When researching biomedical implants to improve their effectiveness and performance, it is crucial to carefully consider their long-term effects on cellular sites. It is worth noting that the small size of these micro/nanocarriers allows them to closely resemble biomolecular and cellular structures, which can potentially lead to certain toxicities. Hence, there are potential risks to both human health and the environment associated with this [138]. C Monge et al. achieved extended survival by enclosing living cells within cross-linked alginate microcapsules. Various polymers have been developed over the years to control the coating's thickness, stability, and permeability. While CVD is effective at making thin, consistent coatings, getting extremely thick coatings (more than a few micrometers) could be challenging with this method. A thick coating may negatively affect the cell response and increase the implant size [139].

## 8 Future Trends in CVD coating of biomedical implants

Self-assembling molecules can arrange themselves regularly and tightly on a metal surface, which is advantageous for polymerization applications. This highly pliable and securely adhered layer might provide advantages in coating adherence, corrosion protection, and mechanical and chemical resistance [140]. Due to their inability to comply with modern coatings standards, natural raw ingredients like oils and resins are no longer commonly used as paint binders. However, Such materials may become more in demand due to biochemical gene alterations [141]**.** Self-cleaning coatings, impervious to oil, dir, and water when applied to various surfaces, are another significant breakthrough. The "lotus effect," which occurs when water and debris cannot penetrate a surface, is used in self-cleaning roof tiles and other hygienic products. Because they are easily damaged by washing and other mechanical processes, artificial lotus surfaces are not expected to be used for vehicle exteriors in the foreseeable future. The use of surface effects, such as shark skin and fur-like films, in the coating of polymers may also be explored [140]. Biomedical implants are being fine-tuned for usage in live creatures' tissues by narrowing their focus and using a modified design to maximize compatibility. Direct surgical remedies are eventually rendered unnecessary by this method. Plastic processing techniques have greatly enhanced the durability of Titanium composite, making it more resistant to scratching, yielding, and fatigue failure in 2CP Titanium [142].

Hassanin et al. [143] reported that a selective laser melting technology has recently made it feasible to achieve controlled medication release from titanium implant surfaces. It is believed that the user may predict and adjust the drug release kinetics via the microchannels and drug-loaded internalized compartments. The topographies have been optimized to obtain a minimal variance in the dimensions of the microchannels and the surface roughness at a range of 1–2 μm, which is dependent on the power of the laser. Even though studies have shown that laser-deposited titanium implants have a longer lifespan, the practical applicability of these implants is currently somewhat restricted. This presents an excellent opportunity to develop an implant-mediated targeted injection system.

Nanotechnology has triggered dramatic changes in many areas of science and technology in recent years. Fabricating far superior surface coatings is possible because cutting-edge techniques leverage nanoscale phenomena. How much of an impact nanotechnology has on coatings will depend on how successfully it guides the development of hierarchical systems that include nanostructures. Tailoring coatings for specific functions will be the focus of future technologies, whether by incorporating easily recognized components into the desired coating or generating unique structures during the coating process [144]. The ability to synthetically produce materials with novel physical, chemical, and mechanical properties has piqued interest in nano-coating. Nano coatings can be designed in a variety of ways, with terms like "nanocomposite" and "nanoscale multilayer" being thrown around [145]. Although tungsten carbide hard metals and their analogs are now considered mature technologies, there has been significant interest in modifying microstructure design to create alternatives to the standard two-phase structure. Changing the phase sizes, shapes, and distributions to create ultra-fine-grained materials has led to significant performance gains in coatings [146].

## 9 Conclusion

The CVD method is a versatile coating technology that can deposit various materials onto biomedical implants. Implants can benefit from CVD coatings, as they have the potential to improve biocompatibility, antibacterial properties, resistance to fouling, and bioactivity. Biomedical implants play a crucial role in modern medicine, offering individuals renewed hope and an improved quality of life. Biocompatibility and durability issues, however, have continued to be a concern, emphasizing the need for innovative solutions. In biomedical engineering, enhancing implant performance is of utmost importance to advance patient care. The paper has emphasized the significant impact of Chemical Vapor Deposition (CVD) on surface engineering, specifically in improving the biocompatibility and longevity of biomedical implants. Our investigation has shown that CVD has the potential to significantly enhance durability, decrease friction, and improve the compatibility of implant surfaces. The longevity of the implants and the reduction of adverse biological responses are greatly enhanced by these advancements, making the implants a safer and more reliable option for patients. The case studies and experimental data presented highlight CVD's practical application and benefits in real-world biomedical contexts.

CVD-based surface engineering offers a robust and adaptable method for enhancing the interaction between biomedical implants and human tissues. With the continuous evolution of research and technology, biomedical implants are set to be propelled forward by integrating CVD techniques. This integration promises to enhance medical treatments, making them more effective and long-lasting. This paper sets the stage for future investigations and developments, promoting ongoing exploration of CVD's complete potential in improving the performance of biomedical implants.

## Data availability Statement

Data used to support the findings of this study are available from the corresponding author upon request.

## Funding

The author(s) declare that no financial support has been received for this article's research, authorship, and/or publication.

## CRediT authorship contribution statement

**Tasfia Saba:** Writing – original draft. **Khondoker Safin Kaosar Saad:** Writing – original draft. **Adib Bin Rashid:** Writing – review & editing, Supervision, Conceptualization.

## Declaration of competing interest

The authors declare that they have no known competing financial interests or personal relationships that could have appeared to influence the work reported in this paper.
